# A multiscale systems perspective on cancer, immunotherapy, and Interleukin-12

**DOI:** 10.1186/1476-4598-9-242

**Published:** 2010-09-15

**Authors:** David J Klinke

**Affiliations:** 1 Department of Chemical Engineering and Mary Babb Randolph Cancer Center, West Virginia University, Morgantown, WV 26506-6102, USA; 2 Department of Microbiology, Immunology & Cell Biology; West Virginia University, Morgantown, WV 26506-6102, USA

## Abstract

Monoclonal antibodies represent some of the most promising molecular targeted immunotherapies. However, understanding mechanisms by which tumors evade elimination by the immune system of the host presents a significant challenge for developing effective cancer immunotherapies. The interaction of cancer cells with the host is a complex process that is distributed across a variety of time and length scales. The time scales range from the dynamics of protein refolding (i.e., microseconds) to the dynamics of disease progression (i.e., years). The length scales span the farthest reaches of the human body (i.e., meters) down to the range of molecular interactions (i.e., nanometers). Limited ranges of time and length scales are used experimentally to observe and quantify changes in physiology due to cancer. Translating knowledge obtained from the limited scales observed experimentally to predict patient response is an essential prerequisite for the rational design of cancer immunotherapies that improve clinical outcomes. In studying multiscale systems, engineers use systems analysis and design to identify important components in a complex system and to test conceptual understanding of the integrated system behavior using simulation. The objective of this review is to summarize interactions between the tumor and cell-mediated immunity from a multiscale perspective. Interleukin-12 and its role in coordinating antibody-dependent cell-mediated cytotoxicity is used illustrate the different time and length scale that underpin cancer immunoediting. An underlying theme in this review is the potential role that simulation can play in translating knowledge across scales.

## Introduction

Therapies targeting particular molecules relevant in the pathogenesis of cancer promise efficacy in stratified patient groups with minimal side effects. Breast cancer is a prime example where a molecular therapy - trastuzumab - has been shown to have remarkable efficacy in patients with tumors that overexpress one of the epidermal growth factor (EGF) receptors, ErbB2 [1,2]. In 25-30% of breast cancer patients, the ErbB2 receptor is overexpressed and is correlated with a poor prognosis [3]. Trastuzumab is a monoclonal antibody that specifically targets the ErbB2 receptor and blocks the interaction of ErbB2 with other members of the EGF receptor family [4,5]. Trastuzumab halts abnormal cell proliferation by decreasing ErbB2 expression through sequestering it in endocytic vesicles, resulting in receptor degradation [6]. Yet, one of the persistent challenges in cancer research is understanding why patients who overexpress these targeted proteins either do not respond at all or ultimately become resistant to the therapy. For instance, only 12-34% of patients that overexpress ErbB2 respond to trastuzumab by itself, and then only for a mean period of 9 months [1,7]. The fact that all patients eventually develop resistance to trastuzumab represents an important, and poorly understood, clinical problem (e.g. [8,9]). Moreover, monoclonal antibodies form one of the largest classes of molecular targeted therapies for cancer [10]. While molecular targeted drugs attack a single target, it is increasingly evident that a multitude of factors (e.g., immunological bias, genetic predisposition, and oncogenic changes) contributes to cancer etiology. Using the immune system as a source of patient-generated antibodies to provide a similarly selective but also adaptive therapy has intrigued immunologists and cancer biologists for decades [11]. In the recent decade, the concept of cancer immunoediting holds renewed promise following numerous studies on human immunodeficiencies that provide support for the role of lymphocytes (e.g., T, NK, and NKT cells) and cytokines in regulating primary tumor development [12]. Adjuvants, such as Interleukin-12, also hold promise for augmenting antitumor immunotherapy [13].

Interleukin-12 (IL-12) is an important immune regulatory cytokine that exerts potent antitumor activity and a member of a small family of heterodimeric cytokines [14,15]. In the literature, IL12 implicitly refers to a 75-kDa heterodimer that is formed by the disulfide-linkage of two independently regulated gene products: a 40 kDa (p40) subunit and a 35 kDa (p35) subunit [16]. The p40 subunit, as a homodimer (IL12(*p*40)_2_) or monomer (IL12p40), can also bind to the IL-12 receptor resulting in interactions that antagonize IL12p70 binding both in mice [17,18] and humans [19]. The bioactivity of IL-12 is due to the competitive binding of all isoforms with the IL-12 receptor [20]. In the peripheral tissues, IL-12, originally called Natural Killer Cell Stimulating Factor, enhances the ability of NK cells to lyse target cells, a mechanism exploited for tumor immunotherapy [21]. As an adjuvant, IL-12 promotes NK-cell mediated killing of HER2-positive tumor cells in patients treated with trastuzumab [22-24]. Yet despite the sincere efforts of many to understand the complicated relationship between cancer and the immune system, translating the therapeutic potential of immunotherapies observed in vitro and in animal models to the clinic has been difficult [25].

One of potential sources for this difficulty has been how we have predominantly approached this problem. "Divide and conquer" has been used to describe the predominant mode of scientific inquiry in the medical sciences [26]. The underlying assumption is that understanding the behavior of a complicated system can be achieved by deconstructing the system into more fundamental components and characterizing the behavior of the components. In studying the fundamental components in isolation, we may miss collective interactions that are important for understanding how the integrated system works. In addition, this reductionist approach towards scientific inquiry also spawned subdisciplines that focus on specific aspects of biological systems. For instance, the study of protein structure and folding typically falls under the purview of biophysics, the study of metabolic and signaling pathways falls under the purview of biochemistry, and the study of emergent behavior of populations of immune cells to biochemical cues falls under the purview of immunology. The engineering disciplines have taken a different approach towards understanding natural and synthetic systems. For instance, chemical engineering has a rich history where theory and mathematics provide a framework for analyzing, designing, and controlling reacting systems [27,28]. One of the unifying concepts in the discipline is that theory and mathematics can be extended using simulation. Using simulation, engineers predict the behavior of complicated systems using knowledge of system components and theories (e.g., transport phenomena and chemical kinetics) that describe how we expect the components to interact. These predictions are then tested experimentally to ask the question: is our incomplete knowledge of the system components sufficient to reconstruct the behavior of the system? In the process, a more fundamental question is asked: is this system complicated (i.e., components interact via defined rules that we can characterize in isolation) or is it complex (i.e., the behavior of components is an emergent behavior that can only be characterized by studying the integrated system)? Collectively, this process is a knowledge generating activity [29]. This process also helps manage uncertainty: do we understand the system sufficiently to make a decision or do we need to gather more data. From this perspective, research activities associated with the disciplines of engineering and basic medical sciences represent contrasting modes for acquiring knowledge about systems (i.e., reconstruction versus deconstruction). The objective of this review is to describe methods used in engineering to study systems and to analyze cancer immunotherapy from an engineering perspective, using IL-12 as an illustrative example.

## Systems Analysis and Identifying Scales

When presented with a complex problem, such as developing a novel immunotherapy, a common problem-solving approach is to first identify the important components whose interactions define system behavior. Advances in molecular biology during the twentieth century provided experimental tools to identify the individual components of complex biological systems [30]. Once identified, the function of these components and their interactions can be characterized. In engineering, this process is called systems analysis [31].

Knowledge obtained by systems analysis is coupled to the experimental techniques that scientists use to probe systems and the computational tools that are used to interpret those experimental observations. One of the particular techniques used in systems analysis is to identify the different time scales that underpin the response of a dynamic system (i.e., a time scale analysis) to an abrupt change in environmental conditions. A time scale analysis aids in simplifying the response of a system by parsing system components and their corresponding dynamics into different kinetic manifolds (e.g., [32]). The evolution in the system is constrained by the slow variables (i.e., the slow kinetic manifold) while the fast variables (i.e., the fast kinetic manifold) exist at a pseudo-equilibrium. Moreover, variables that exhibit time scales significantly longer than the time scale over which the system has been observed can be considered stationary (i.e., a stationary manifold). This phenomenon related to separating time scales has been termed the slaving principle [33]. From observed differences in time scales, we can infer that the important components that regulate the system dynamics correspond to the slow kinetic manifold. Components that correspond to a stationary manifold do not need to be represented explicitly as their contributions can be lumped into appropriate rate parameters. Components that correspond to a fast kinetic manifold can be described using equilibrium relationships (i.e., experimentally measurable equilibrium dissociation constants rather than kinetic rate parameters). Time scale analysis is a classical technique used to identify key enzymes that control flux within [34] and quantify hierarchical relationships among elements of a complex metabolic network [35].

Similarly, the distance over which components interact (i.e., a characteristic length scale) can also be identified. In systems where components move (i.e., diffuse) and can be transformed (e.g., degradation of a protein ligand upon binding to a cell), a characteristic length scale can be defined as a ratio between the rate parameters for diffusion and reaction [36]. This approach has been used to explain the inverse relationship between penetration of therapeutic antibodies into tumor spheroids and the affinity of the antibody to the tumor antigen, called the "binding site barrier" [37]. The effective depth of penetration, *λ*, is defined as:

(1) $$\lambda \text{ =}\sqrt{\frac{D\text{· }{\left[Ab\right]}_{o}}{k_{e}\text{ · }{\left[Ag\right]}_{o}}}$$

where *D *is the effective diffusion coefficient for antibody penetration into tumor spheroids, [*Ab*]*_o _*is the concentration of antibody in the tissue, *k_e _*is the rate constant for the catabolism of antibody upon binding to the corresponding tumor antigen, as represented by the average concentration of the tumor antigen within the tumor ([*Ag*]*_o_*) [38]. Note that the length scale in this example, *λ*, is a function of the rate parameter, *k_e_*. The rate parameters are also used to estimate time scales. This highlights the direct relationship between time and length scales.

**Figure 1:**
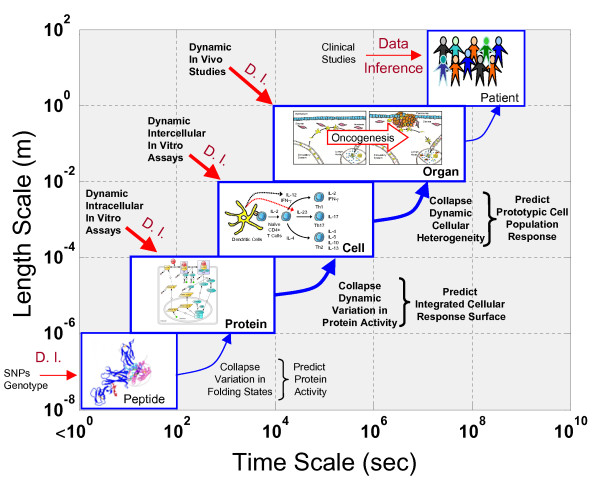
**An overview of the multiple time and length scales involved with understanding cancer immunotherapy**. Five subsystems are shown that each represent a limited range of time and length scales and are named after the basic functional unit: peptide, protein, cell, organ, and patient. Within each subsystem, knowledge about behavior of a particular subsystem is inferred from observed data, as depicted by the red arrows, and prior information, as depicted by the blue arrows that enter each subsystem box. Each experimental assay has an intrinsic length and time scale and thus inform the corresponding subsystem. Prior information for interpreting data within a subsystem can be obtained from a summary of the dynamics of subsystem with shorter time and length scales. This summary of the dynamics may take the form of equilibrium values or population-based averages.

Cancer is a complex multiscale system that spans multiple time (e.g., milliseconds to years) and length scales (e.g., nanometers to meters) [39]. In studying cancer, we implicitly focus on a narrower range of scales to ask more focused questions: how do immune cells process information at the molecular level, how does the immune system shape tumor cell populations, or are there genetic differences associated with clinical response to a cancer immunotherapy. This implicit partitioning of a multiscale system into a series of subsystems that are constrained to a narrower range of time and length scales aids in reducing the complexity of the problem. A set of subsystems that are relevant to cancer immunotherapy include the peptide, protein, cell, organ, and patient levels, as depicted in Figure 1. Given the direct relationship between time and length scales, the subsystems are placed along the diagonal in this diagram. The labels correspond to the basic component unit within each of subsystem. Within each of these subsystems, knowledge regarding the behavior of components within a particular subsystem is inferred from observed data and prior information. Following from the "slaving principle," information passes from subsystems that exhibit shorter time and length scales to subsystems that exhibit longer time and length scales. This can be represented as the trafficking of information from the bottom upwards, as highlighted by the blue arrows in Figure 1. For instance, the dynamic distribution in conformational states at the peptide level is summarized in terms of a protein-protein interaction energy (i.e., protein activity). The activity of a protein provides prior information for higher time and length scales. Absent any alterations in protein structure (e.g., SNPs or mutations), the energetics of protein-protein interactions that contribute to the existence of edges within a canonical signaling network are typically assumed to be conserved across systems. How a cell processes information via a signaling network is then determined from observed measurements in changes in expression or activity of an intermediate signaling protein, given known protein-protein interactions. In modeling cell level behavior, it may not be necessary to incorporate details regarding the dynamics of a signaling network nor to incorporate protein-folding dynamics. It may be sufficient to represent signaling networks as a collection of rules that relate extracellular signal to cellular response (i.e., an integrated cellular response surface). These rules may represent simple input - simple output relationships (i.e., how a change in a single cytokine influences cellular proliferation) or they may represent multiple input - multiple output relationships to account for context-dependent behavior (i.e., how changes in multiple cytokines collectively influence cellular survival and cytokine production). In the following sections, we will expand on this multiscale concept by focusing on Interleukin-12 and its role in coordinating antibody-dependent cell-mediated cytotoxicity.

## The Peptide Level

Cellular response to extracellular stimuli is governed by protein-protein interactions that allow the transfer of information from the cell membrane to the nucleus and back [40]. Proteins interact through functional motifs that characterize the affinity and specificity for a particular motif-motif interaction [41]. Within this multiscale hierarchy, the peptide level focuses on identifying changes in the protein structure that redistribute the energetic states of a system to prefer different conformations [42]. When two proteins interact via motifs, the distribution in energetic states of the protein complex reaches an equilibrium distribution within seconds and may propagate beyond the motif-motif interaction region. The equilibrium distribution in states characterizes the affinity for a particular protein-protein interaction. Somatic mutations or germline single-nucleotide polymorphisms in the coding region of genes alter the primary protein structure, resulting in a different affinity for protein-protein interactions that contain the mutated protein (e.g., [43]). Experimentally, the binding affinity for motif-motif interactions can be measured using high-throughput in vitro methods [44,45]. The energetics for motif-motif interactions measured in vitro may not correspond to the actual binding affinities of two proteins within a cell that interact through a particular motif pair. Macromolecular crowding or other structural aspects of the proteins may influence the absolute value of the binding affinity. However, the relative differences among the different motif-motif interactions do predict which proteins become activated upon direct interaction with receptor tyrosine kinases [46]. Alternatively, the distribution in energetic states of a protein can be obtained using simulation, as summarized by [47]. Simulation or high-throughput experimental methods can both be used to identify how alterations in the amino acid sequence alter the structure of a protein. Thus, the objective of this level would be to infer protein-protein interaction strength based upon data that describes changes in genotype.

A series of genome association analyses have identified polymorphisms associated with proteins involved in the IL-12 signaling axis. These polymorphisms are typically identified as they correlate with different phenotypes within a clinical population. The phenotypes may be directly (e.g., oncogenic) or indirectly (e.g., alter tumor immunosurveillance) related to cancer. In particular, genetic mutations in IL-12p40 and one component of the IL-12 receptor, IL-12R*β*1, have been observed in patients with recurrent mycobacterial disease [48,49]. Heterozygous mutations in the other component of the IL-12 receptor, IL-12R*β*2, have been reported in atopic patients that correlate with a reduction in STAT4 phosphorylation, the central transcription factor in the IL-12 pathway, and IFN-*γ *production in response to IL-12 stimulation [50,51]. A single point mutation (Val617Phe) in the JAK2, a Janus Kinase that forms a complex with IL-12R*β*2, associates with myeloproliferative disorders [52], promotes the constitutive activation of the kinase, and enables the enzyme to escape negative regulation by SOCS3 [53]. In contrast, mutations that impair kinase activity in TYK2, a member of the Janus Kinase family that interacts with IL-12R*β*1, have been associated with reduced IL-12 responsiveness [54]. Association of coding single nucleotide polymorphisms (SNPs) within the Tyk2 gene with disease in humans has also been identified [55,56]. A reduced response to IL-12, similar to an increase in atopy and susceptibility to mycobacterial disease, is an indication for reduced cell-mediated cytotoxicity, an important effector mechanism for tumor immunosurveillance. In principle, an understanding of how genotype influences protein-protein interaction strength provides prior information for the next level: the Protein level. However, the structural implications of many of these mutations remain unclear. Identifying the physiological implications of SNPs is also difficult due to the overlapping roles that the intracellular signaling proteins play in other signaling pathways. For instance, TYK2 plays a role in IFN-*α *[57] and IL-23 [58] signaling, in addition to IL-12 signaling. Longer time and length scales provide additional perspectives for addressing these questions.

## The Protein Level

The next larger time and length scale focuses on interactions between proteins that occur within the cell. The collective protein-protein interactions form networks, such as metabolic and signaling networks. The structure (i.e., topology) of these networks is described by a series of nodes and edges. The nodes are the individual proteins and the edges, in the case of signaling networks, correspond to the velocity of information flow due to protein-protein interactions. The topology of signaling networks may be inferred from in vitro assays that measure changes in the intracellular state of signaling proteins in response to a suite of stimuli using Bayesian computational methods [59]. Alternatively, canonical pathways are proposed that summarize the collective scientific evidence in support of the topology of a particular signaling network (e.g., [60] and the KEGG PATHWAY database: http://www.genome.jp/kegg/pathway.html). In the literature, these networks are frequently represented as qualitative cartoons that illustrate simple linear "bucket brigades," where information is passed from one protein to another [61]. However, cellular signaling networks have evolved to have complex characteristics, including redundancy (whereby signals are dispersed among multiple pathways) and complex feedback loops (whereby signals are amplified or dampened as they pass through a particular pathway) [62]. As an illustrative example of this complexity, consider the IL-12 signaling network.

**Figure 2:**
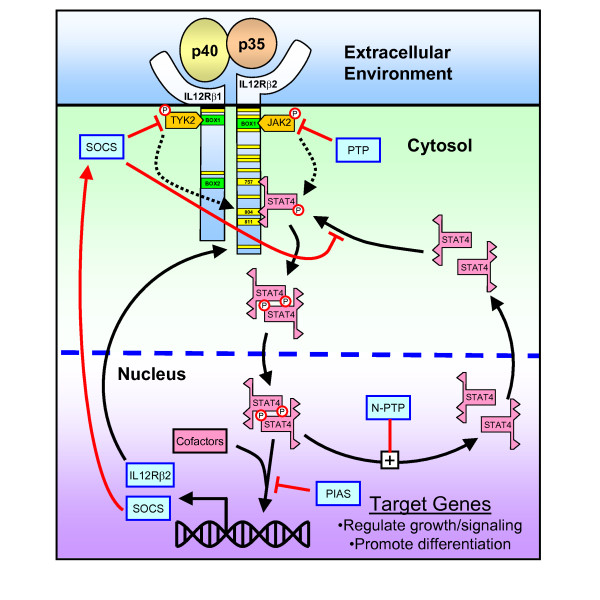
**A schematic diagram of the flow of information from the extracellular environment to the expression of target genes in the nucleus by the canonical IL-12 signaling network**. These signaling networks originate at the cell membrane following the activation of dimers of the cytokine receptors, such as IL12R*β*1-IL12R*β*2. The yellow bars on the IL12R*β*1 and IL12R*β*2 receptors indicate the particular tyrosine residues within the intracellular portions of the receptors. In the mouse, STAT4 interacts primarily with the tyrosine residues Y_757_, Y_804_, and Y_811 _on IL-12R*β*2. The green bars indicate the BOX motifs that interact with the kinases: TYK2 and JAK2. The orange boxes correspond to canonical Janus Kinases, TYK2 and JAK2, that interact with the IL-12 receptor. Key signaling proteins within individual pathways are shown. The red lines indicate protein-protein interactions that negatively regulate this signaling network.

Cellular response to IL-12 occurs via one member of the canonical Janus kinase (JAK) and signal transducer and activator of transcription (STAT) family of signaling pathways [63]. Signal transduction originates with the IL-12 receptor, a member of the type 1 cytokine receptor family and comprised of two subunits: IL-12R*β*1 and IL-12R*β*2. These receptor subunits lack intrinsic enzymatic activity and require association with specific Janus kinases, JAK2 and TYK2, to transmit cellular signals. Binding of a natural ligand to an IL-12 receptor precipitates a series of biochemical events: the receptor changes conformation, the tyrosine residues on the receptor become phosphorylated by receptor-associated Janus kinases, signaling proteins associate with the activated receptor (e.g., STAT4), and the signaling proteins in turn become phosphorylated. In the IL-12 signaling network, phosphorylated STAT4 translocates to the nucleus to promote the transcription of various response genes. A subset of these signaling pathways that lead to different cellular behaviors is depicted in Figure 2.

While the canonical JAK-STAT pathway seems relatively straightforward, various positive and negative regulatory pathways modulate the strength and duration of signaling. As effective signaling via the IL-12 pathway requires the expression of IL-12R*β*2, phosphorylated STAT4 promotes the upregulation of the IL-12R*β*2 subunit [64-66] creating a positive feedback loop. A predominant pathway for negative feedback regulation of IL-12 signaling is via the family of Suppressor of Cytokine Signaling (SOCS). Specifically, SOCS1 inhibits IL-12 signaling [67,68] and SOCS3 negatively regulates IL-12 signaling by blocking the binding of STAT4 to the IL-12R*β*2 subunit [69]. Message for both SOCS1 and SOCS3 increases in IL-12-stimulated peripheral blood T cells [70]. However, the mechanism by which SOCS proteins regulate cytokine-receptor signaling remains unresolved [63]. The current model for SOCS regulation of the JAK/STAT signaling is that the E3 activity of the SOCS protein targets the substrate for ubiquitination and subsequent proteosomal degradation [71]. In contrast, genetic studies suggest that the SH2 domain of the SOCS protein blocks cytokine-receptor signaling by itself [69]. In addition, the protein inhibitors of activated STATs (PIAS) (a.k.a., SUMO) are also negative regulators of cytokine signaling [72,73]. In particular, PIAS inhibits IL-12 signaling by sequestering STAT4 and thereby inhibiting STAT4-dependent gene transcription [74].

**Figure 3:**
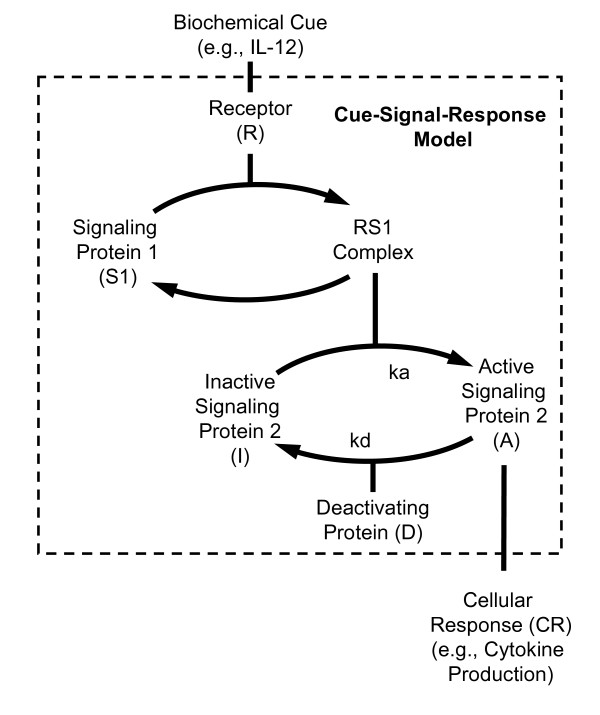
**A conceptual model of the flow of information within an intracellular signaling network**. Biochemical cues initiate a cellular response by interacting with receptors. Cellular receptors modify intermediate signaling proteins via a cascade of activating and deactivating events. Changes in activity of these intermediate signaling proteins ultimately regulate cellular response. In this two level cascade, an activated receptor (R) interacts with signaling protein 1 (S1) to form a multi-protein complex (RS1). The activity of signaling protein 2 is determined by the balance between activation and deactivation rates. The activation and deactivation rates are related to the abundance of the RS1 and deactivating protein (D), respectively. Cellular response is proportional to the activity of signaling protein 2.

As illustrated by the IL-12 signaling example, many of the molecular players in the various signaling pathways are known. However, the regulatory roles that individual proteins play at specific points in time and in particular systems are largely unknown [75]. It is precisely in this situation that mathematical models are most helpful [39]. These models are typically based upon theories that are used to describe how proteins interact. For example, the transfer of information within intracellular signaling networks has been described in terms of a cascade of activating (e.g., kinase action) and deactivating (e.g., phosphatase action) events that modify intermediate signaling proteins [76] (see Figure 3). Within a level of this cascade, the steady state activation of a signaling protein (A) is described by:

(2) $$A\text{ =}\frac{S\text{2 · }RS\text{1}}{\frac{kd\text{·}D}{ka}+RS\text{1}},$$

where *S*2 is the total concentration of signaling protein in both active (*A*) and inactive (*I *) conformations, *RS*1 is the concentration of activating protein complex, *D *is the concentration of deactivating protein, and *ka *and *kd *are the rate constants associated with activating and deactivating proteins, respectively [77].

Cellular response is proportional to the abundance of *A*. While changes in peptide structure alter the rate constants, changes in abundance of any of the participating proteins (e.g., *RS*1, *S*2, and *D *in Equation 2) can also influence cellular response to a particular biochemical cue. These changes in protein expression within a cell are assumed to occur quicker than changes in cell populations and therefore limit the range of relevant timescales. Research questions at the protein level focus on two aspects: 1) how genetic variation influences the flow of information within a signaling pathway and 2) how proteins are dynamically regulated to shape cellular response. In the following paragraphs, each of these aspects will be discussed separately.

As suggested by the theory encoded in equation 2, changes in the expression of proteins involved in the IL-12 signaling network will alter the cellular response to IL-12. Similar to coding polymorphisms described in the Peptide section, polymorphisms in untranslated regions of proteins involved in the IL-12 signaling axis have been identified in genome association studies. Alterations in the genome in untranslated regions can affect the expression of genes and their corresponding proteins. For instance, a recently discovered mechanism for posttranscriptional regulation of gene expression is via miRNAs [78].

Untranslated regions (UTR) of mRNA provide binding sites for regulatory miRNAs. Shortened 3'UTRs are associated with oncogenic transformation in cancer cell lines, a loss of miRNA target sites, and an increase in expression of the corresponding proteins [79]. While no polymorphisms have been identified yet, miRNA have been associated with the IL-12 signaling network including miR-21 that regulates mIL-12p35 expression [80], miR-135a that regulates JAK2 expression [81], and miR-155 that regulates SOCS1 expression [82]. These miRNA may represent regulatory components of a signaling-dependent translational control structure that influences the flow of information within the IL-12 pathway. While not specifically associated with miRNAs, a polymorphism in the 3'UTR of the IL-12p40 gene has been associated with a reduction in plasma IL-12p40 [83,84] and an increase risk for carcinoma [85,86], lymphoma [83], and glioma [84]. In the 5' regions, single nucleotide polymorphisms in the 5' flanking region of the IL-12R*β*2 gene is associated with aggressive periodontitis [87]. In addition, SNPs in the non-coding regions of the STAT4 [88] and IL-12R*β*2 [89] genes have been associated with an increased risk for autoimmunity. SNPs in the non-coding regions of Tyk2 associate with increased risk for inflammatory bowel disease [90].

Besides single-nucleotide polymorphisms, other genetic and epigenetic changes modulate protein expression. Chromosomal translocations may switch the corresponding promoter to a more active one or change the regulation of gene expression [91]. Structural genomic variation, with the majority smaller than 10 kb, is a major contributor to phenotypic variation within the normal human genome [92,93]. The highest proportion of genes affected by the identified variants modulates cellular response to extracellular signals (e.g., receptor signaling networks). One of the functional effects of structural genomic variants is a change in the level of expression of gene products for a given transcription signal. Alterations in DNA copy number variants have also been observed in solid tumors [94]. Epigenetic mechanisms also regulate gene expression and promote oncogenesis [95]. Epigenetic silencing of the IL-12R*β*2 gene via DNA methylation has been observed in chronic B-cell malignancies compared to normal B-cells [96] and primary lung adenocarcinomas [97].

The theory encoded in equation 2 can be extended using mathematical models. To create a mathematical model, one must first specify the causal relationships among the interacting proteins involved in a signaling network (i.e., the network topology). Similar to Bayesian networks, ordinary differential equation (ODE)-based mathematical models provide a computational framework for expressing the current knowledge regarding the topology of a signaling network. Historically, the topology of a reaction network has been assembled manually through the judicious use of simplifying assumptions (e.g., [98-100]). These manually assembled networks have provided insight into many signaling pathways [62]. However, the implicit assumptions required for manual assembly of reaction networks impose bias and limit wider application [101]. One of the advances in the field of reaction pathway analysis has been the creation of algorithms that automatically generate reaction networks using formalized descriptions of molecular transformations [102,103]. Algorithms that automate model construction allow the researcher to focus on interpreting the biochemistry described by the model rather than on its tedious assembly.

Graph theory is a useful mathematical framework that facilitates constructing a reaction network among reacting species [104] and provides the fundamental basis for these algorithms. The generality of the approach lends itself to representing different reacting systems with minimal modification to the algorithm. Examples of applications include reaction networks that contain hydrocarbons [105], immobilized binding sites [106], and multi-state proteins [107-111]. Representing multi-state proteins as a collection of functional motifs [41] is a key concept that enables applying this computational approach to signaling networks. Reaction networks, like cell signaling networks, can be constructed based upon the systematic application of "rules" that provide constraints on the formation and destruction of motif-motif "bonds."

Application of the rules to reacting species can create reaction networks that exhibit combinatorial complexity [112], leading to a combinatorial explosion in the number of unique species represented in the model [111]. However, computational tools have been developed to prune the reaction network based upon specific criteria and to facilitate intuitive interpretation of model behavior [105,113]. Once the network topology has been specified, ODE-based models provide quantitative predictions following the specification of initial conditions for the model variables and of values for the reaction parameters. Initial conditions can be estimated from protein expression measurements and reaction parameters can be estimated using protein-protein affinity data, dynamic calibration data, and thermodynamic constraints (see [114] as an example).

Unlike Bayesian networks, ODE-based models can be used to infer how proteins dynamically regulate the flow of information down different branches with a signaling network from observed data [115]. However, the ability of a particular mathematical model to describe a system of interest, analogous to experimental studies, must include a statement of belief. Belief derived from a mathematical model is expressed commonly in terms of a single point estimate for the predictions, obtained from the set of parameters that minimizes the variance between model and data [116]. Given that a model constrains the set of possible states of the system, it is essential to provide an estimate of the uncertainty associated with the model predictions given the available data. The use of single point estimates is a frequent point of contention in the use of mathematical models, as the values for many of the parameters are not precisely known. The logical argument is that if the uncertainty in values of the model parameters is high, then the uncertainty in the model predictions should also be high. However, recent developments in methods for Bayesian model-based inference address this concern.

A Bayesian view of statistics is a mathematical expression of our beliefs [117]. Beliefs are established based upon the observation of data and the interpretation of that data within the context of our prior knowledge [118]. Mathematical models provide a quantitative framework for representing prior knowledge of the detailed biochemical interactions that comprise a signaling network. The unknown parameters of the model are calibrated against the observed network dynamics. Given the calibration data and the postulated model, the uncertainty in the model predictions can be obtained using an empirical Bayesian approach for model-based inference [115,119]. In essence, these methods are computationally intensive methods that randomly walk within parameter space (i.e., a Monte Carlo approach). New steps in parameter space extend the walk. A potential new step is evaluated by comparing the model predictions obtained using the parameter values of the new step against the available data. The model predictions for the new step are only compared against the current step in the random walk (i.e., it is a Markov Chain). The similarity between the model predictions and the available data correspond to the likelihood for including the potential new step in the on-going walk. High agreement between model predictions and the available data has a high likelihood for inclusion in the on-going walk while low agreement has a low likelihood for inclusion. When the random walk has sufficiently traversed the parameter space as to provide consistent model predictions, the Markov chain is considered to be converged. The collection of model predictions contained within the converged segment of the Markov chain provide an estimate of the uncertainty in the model predictions that reflects both the specific data at hand and the uncertainty in the values of model parameters. This approach has been used to infer the strength of different positive- and negative-feedback mechanisms within the IL-12 signaling network in naïve CD4+ T cells obtained from Balb/c mice [120]. One of the conclusions of this work is that not all of the parameters need to be precisely defined for the model to provide narrowly distributed predictions. In other words, we can be highly confident in our ability to discriminate among competing hypothesis regarding the flow of cellular information, as encoded in a mathematical model, despite the underlying uncertainty in the model parameters. Ultimately, understanding the dynamic regulation of signaling networks will enable one to map biochemical cues onto cellular response in the form of deterministic cellular rules. This mapping of biochemical cues to cellular response provides prior information for the next level: the Cell level.

## The Cell Level

**Figure 4:**
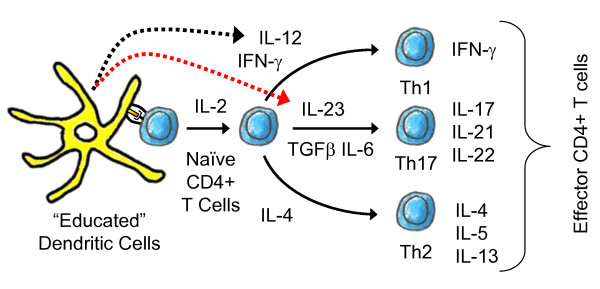
**An overview of the cytokines involved CD4+ T helper cell expansion and polarization**. Naïve CD4+ T cells can differentiate into one of three lineages of effector T helper (Th) cells - Th1, Th2, and Th17 - following signaling via the T cell receptor and co-stimulatory receptors. The effector Th cell populations are defined based upon their cytokine production profile and perform distinct immunoregulatory functions. Th1 cells assist in regulating antigen presentation and cell-mediated immunity. Anti-parasite and humoral immunity is regulated by the cytokines produced by Th2 effector cells. The cytokines produced by the Th17 subset regulate an inflammatory response.

At the cell level, IL-12 is a paracrine cytokine that provides a critical interface between innate and adaptive immunity [15]. The time associated with an evolving cell population within a particular organ (e.g., antigen-induced expansion and polarization of naïve CD4+ T cells) and the spatial range of paracrine action provide the time and length scale context for this level. As summarized by Figure 4, IL-12 plays a critical role within secondary lymphoid organs in promoting anti-tumor immunity. Sufficient and sustained signaling [70] by IL12p70 through the IL-12 signaling network leads to polarization of naïve CD4+ T cells into a Th1 phenotype [121]. Polarization into a Th1 phenotype promotes anti-tumor immunity via cytokine help for CD8+ T cell expansion and switching B cell antibody production to isotypes, such as IgG2a in the mouse, that enhance antibody-dependent NK cell-mediated cytotoxicity [122].

Mature dendritic cells (DCs) are some of the most prolific producers of IL-12 and play a critical role in regulating the immune response [123,124]. Another member of the IL-12 family, IL-23 has been associated with promoting polarization towards and expansion of a Th17 subset [125,126] and is produced by DCs [127,128]. However, the role of Th17 cells in shaping anti-tumor immunity is still unclear [129]. Another regulatory cytokine, IL-4, promotes polarization towards a Th2 phenotype [130]. In general, it is thought that a Th2 bias correlates with tumor tolerance (e.g., [131]). The association of different regulatory cytokines with different T helper cell subsets, as illustrated in Figure 4, summarizes cell level events that regulate T helper cell polarization in the secondary lymphoid organs. However, biochemical cues play different roles in different organs due to direct action of biochemical cues on the cells that traffic to specific organs. In contrast to its role as a regulatory cytokine in T helper cell polarization, IL-12 enhances the ability of NK cells to lyse antibody-coated target cells in the peripheral tissues [24]. This dual role, as activator of NK cells and as promoter of Th1 polarization, motivates using IL-12 as an adjuvant for antibody-based tumor immunotherapy [23].

In addition to understanding the paracrine action of biochemical cues, the cell level also focuses on understanding how organ-specific system behavior (e.g., a primary immune response within a secondary lymphoid organ) emerges from the collective action of cell populations that exhibit slight variation in phenotype. In addition to the regulatory cytokines, T cell responses are also regulated by antigen recognition. Collectively, the frequency of T cells that recognize specific epitopes influences the quality of immune response [132,133]. In addition, heterogeneity in T cell commitment may be responsible for the observed plasticity in the immune polarization to the recognized epitopes [134]. On the tumor side, cellular heterogeneity within cells of a tumor has been recognized for several decades [135]. More recently, genomic techniques have provided insight into the early genetic heterogeneity in disseminated tumor cells compared to cells of the primary tumor [136]. However, measuring the evolution in cellular heterogeneity in clinical samples has been a particular challenge [137].

In cell populations that carry the same genes, cellular heterogeneity can be attributed to two primary sources. First, variability in cellular response can be attributed to heterogeneity in expression and activity of proteins involved in the signaling pathways that facilitate cellular decision-making. This heterogeneity is observed in similar cell populations using polychromatic flow cytometry [138]. In addition, the regulatory proteins that facilitate this transfer of information may be expressed in low abundance [139]. As the concentration of interacting regulatory proteins decreases, the discrete nature of protein-protein interactions becomes more apparent and gives rise to random fluctuations in the information transfer process. Thus, even in cells that exhibit the same number of regulatory proteins, cellular responses to the same stimulus may be phenotypically different [140]. These internal sources of cellular variability are defined as "intrinsic" sources.

Second, variation in the local microenvironment that surrounds each cell within a population may contribute to variations in collective cellular response. The sources of cellular heterogeneity that are external to the cell are defined as "extrinsic" sources. Experimental approaches, such as 3-D cell culture, provide methods to explore how these extrinsic sources influence cellular response [141]. While the study of intrinsic sources of heterogeneity has been studied by several groups (e.g., [142,143]), extrinsic sources may have greater impact on cellular variability than intrinsic sources, due to the simultaneous influence of external cues on many signaling pathways within a cell [144]. Collectively, these external cues reflect the composition of stromal and immune cells within the tumor microenvironment. The composition of immune cells the tumor microenvironment correlate with clinical response to tumor immunotherapy. For instance, overall survival in Head and Neck Squaemous Cell Carcinoma patients treated with IL-12 correlate with an increased presence of CD56^+ ^NK cells within the primary tumor, irrespective of IL-12 treatment [145]. In addition, impressive infiltration of CD20^+ ^B cells around the tumor was observed in some IL-12 treated patients. Understanding how an immune response is coordinated leads to the next levels: the organ and patient levels.

## The Organ Level

Anti-tumor immunity is a dynamic process coordinated via cellular interactions distributed in time and space. The organ level represents the time and length scales associated with an adaptive immune response. The time associated with developing and maintaining immunological memory is the primary focus of this timescale and spans days to years. Control of an immune response is distributed among different organs of the body, whereby specific cells perform different functions in each organ and the migration of cells between organs enables the transfer of information. As an example of a cell type that conveys information among organs, consider the dendritic cell.

**Figure 5:**
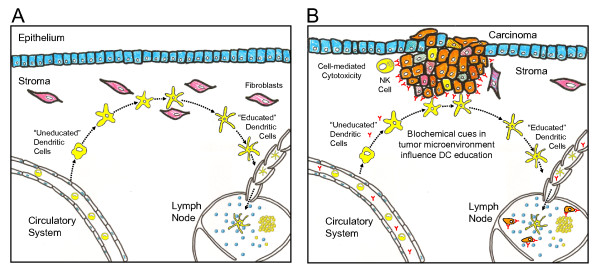
**A schematic diagram of the multi-organ process involved in immunosurveillance that becomes dysregulated in cancer**. (A) Immature dendritic cells are recruited into peripheral tissues from the circulation. While in the peripheral tissues, biochemical cues within the tissue microenvironment educate immature DC. "Educated" mature DC downregulate tissue homing and upregulate chemokine receptors that promote DC emigration to the draining lymph node. Within the draining lymph node, mature DC present antigen, express costimulatory molecules, and secrete cytokines that influence T cell activation and polarization. The particular profile of cytokines secreted by mature DC is imprinted on immature DC while being educated in the peripheral tissues. (B) The presence of an epithelial tumor alters the profile of biochemical cues used to educate immature DC within the tissue microenvironment. In addition, the presence of metastatic tumor cells within the draining lymph nodes may interfere with the role that mature DC play in orchestrating an immune response. Therapeutic antibodies promote antibody-dependent cell-mediated cytotoxicity. Increased cell death by the carcinoma provides an additional source of tumor-associated antigens for immature DC to present in the draining lymph node.

As the sentinels of the immune system, dendritic cells (DCs) play an important role in initiating and maintaining T cell responses, such as T-helper cell polarization [146,147]. The precise role played by DC in *de novo *activation of T cells is the culmination of a series of steps distributed across both space and time. These sequential steps, as shown graphically in Figure 5, include the recruitment into the peripheral tissue, capture of antigen and "education" in a peripheral tissue, and trafficking to a draining lymph node. In the process of migrating from the peripheral tissue to a draining lymph node, DCs undergo a series of phenotypic changes in cell surface marker expression that are collectively called DC maturation. Proteins expressed on the cell surface enable a cell to sense and respond to its environment. These dynamic changes in DC proteins indicate that the particular cellular response of a DC to the environmental context is highly dependent on the DC's particular maturational age. Upon arrival to the draining lymph node, mature DC initiate an appropriate T cell response by presenting antigen, upregulating costimulatory ligands, and releasing mediators, such as IL-12.

As recently summarized [148,149], the production of IL12p70, IL12p40, and IL12(*p*40)_2 _by mature DC in the draining lymphoid organ is highly dependent on the cells' cumulative exposure to inflammatory mediators during differentiation and maturation [150] and thus provide a link between the peripheral tissues and lymphoid organs. These studies highlight the difficulty in ascribing biological roles to biochemical cues based upon in vitro studies alone. The simulations suggest that the combination of both IL-4 and IFN-*γ *in the peripheral tissues significantly increases the polarization of naïve CD4+ T cells towards a Th1 phenotype. As was suggested by Hochrein et al. [151], the impact of IL-4 on DC education suggests an indirect promotion of Th1 polarization. In contrast, it is stated frequently that IL-4 promotes the Th2 polarization of naive CD4+ T cells [130]. However, the Th2 polarization potential of IL-4 is based primarily upon the direct action of IL-4 and IFN-*γ *on naïve CD4 + T cells observed in vitro. This result highlights the pleotropic nature of IL-4, whereby the spatial restriction in IL-4 expression may differentially influence CD4+ T cell polarization.

Under normal conditions, cells of the immune system inhibit tumor growth and progression through the recognition and rejection of malignant cells, a process called immunosurveillance. However, the immune system sculpts tumor development by selecting for malignant variants that create an immunosuppressive microenvironment, thereby blocking productive antitumor immunity. This collective process is referred to as cancer immunoediting [12]. This shift in immune behavior from immunosurveillance to immunotolerance to a tumor is shown schematically in Figure 5B. Tumors promote tolerance by producing biochemical cues that suppress immune function, including TGF-*β*, IL-6, IL-10, and prostaglandin E2 [152,153]. Upon metastasis, the biochemical cues secreted by tumor cells can directly interfere with the cellular communication necessary for eliciting an appropriate immune response. For instance, TGF-*β *inhibits the biological activities induced by IL-12 [154] through an undefined mechanism [155]. In addition, IL-6 has been shown to downregulate IL-12R*β*2 expression in primary polyclonal plasmablastic and multiple myeloma cells [156].

While still localized to the primary site, biochemical cues secreted by the tumor can indirectly bias T cell response through their influence on DC education. For instance, many tumors express elevated levels of cyclooxygenase-2, which is essential for the synthesis of prostaglandin E2 (PGE2) [157-159]. PGE2 exhibits cross talk with IL-4 and IFN-*γ *during DC differentiation and maturation such that PGE2 may promote Th2 polarization even in the presence of IL-4 and IFN-*γ *[149]. In vitro, PGE2 has also been shown to modulate characteristics of DC maturation including upregulation of the chemokine receptor CCR7 [160], essential for homing to secondary lymphoid organs, and inhibition of DC differentiation [161]. However, the in vivo significance of these effects of PGE2 on differentiation and maturation has not been demonstrated. The expansion in the diversity of antibodies against tumor-associated antigens highlights the functional role that an integrated immune system can play in cancer remission [162-164]. Cancer immunotherapies can be viewed as a mechanism to induce an adaptive response against tumor antigens [165]. There are multiple points where tumors may interrupt this integrated process. In vitro study may identify protein-level and cell-level mechanisms by which tumors manipulate immunity. However, inferring how these protein-level and cell-level mechanisms combine to influence system behavior from observations obtained at the organ and patient levels is a particular challenge and is one of the most pervasive problems in the analysis of physiological systems [166].

In engineering, this problem is called an identification problem where causal relationships between system components are inferred from a set of input and output measurements [166]. In this context, an input may be antibodies against tumor-specific epitopes and an output may be tumor regression. Many approaches exist for the identification of simple single-input-single-output (SISO) systems. In addition, many experimental studies characterize how isolated components of physiological systems respond to inputs.

**Figure 6:**
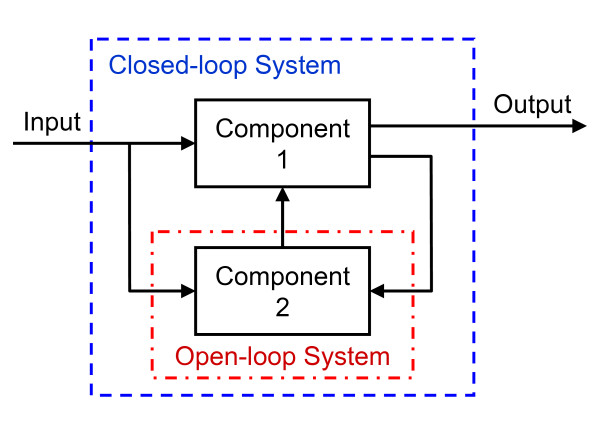
**A schematic diagram of a two-component closed-loop system**. The behavior of a closed-loop system, enclosed within the blue dotted box, is characterized by measurements of variables that provide input to and that reflect the output of the overall system. These variables are depicted as lines that cross the system boundary, depicted by the dotted blue box. The internal variables that are not observed facilitate communication among the system components. Output variables for one component may provide input variables for another component. This internal communication may alter system behavior such that the same system input may result in different system output depending on the internal state of the system. Measurement of internal variables enables characterizing the causal relationships between input variables and output variables for a specific component within an intact system. Ideally, measuring these internal variables reduces complex closed-loop system to a series of connected open-loop systems, as depicted by the red dot-dashed boxes. In an open-loop system, changes in input variables result in a defined response of the system.

However, approaches for identifying causal relationships among components of more complex closed-loop systems, like the immune system, are less well developed. Typically, a closed-loop system is defined as a multi-component system where the output (i.e., response) of one component provides the input (i.e., stimulus) to another component. A schematic diagram of a closed-loop system comprised of two components is shown in Figure 6. Closed-loop systems are particularly challenging, as it is impossible to identify the relationships among components of a system based upon overall input (e.g. peptide-pulsed DC vaccines) and output (e.g. tumor regression) measurements. One of the reasons for this is that changes in the internal state of the system may alter the response of the system to a defined input, such that there is not a direct relationship between overall system input and output. Historically, the causal mechanisms underlying the behavior of closed-loop systems in physiology have been identified via ingenious methods for isolating components within the integrated system (i.e., "opening the loop"). A classic example of this is the discovery of insulin and its role in connecting food intake to substrate metabolism. As insulin is only produced by the endocrine pancreas, the measurement of plasma insulin provides a direct measurement of the communication between food intake and substrate metabolism in the peripheral tissues. The pancreas can then be approximated as a SISO system where the glucose concentration in the portal vein is the input and insulin release into the plasma is the output, as depicted in the Minimal Model for the regulation of blood glucose [167]. Measuring insulin changes in response to changes in glucose provide the basis for partitioning alterations in system response (i.e., diabetes) into deficiencies in insulin production (i.e., type 1 diabetes) and insulin action (i.e., type 2 diabetes). Treatment for diabetes is tailored to the deficiency in component function that exists in the patient.

By opening the loop, a closed-loop system is reduced to a series of connected SISO components. Opening the loop in the context of tumor immunity may refer to the dynamic measurement of internal states of the DC subsystem *in vivo *including: blood precursor populations, biochemical cues produced in the tumor microenvironment, and characteristics of DC that traffic to the draining lymph node. In conjunction with knowledge of the T cell repertoire, this would enable one to develop a more quantitative view of tumor escape mechanisms (i.e., how differences in central repertoire selection, local lymph node cytokine production, and DC education collectively influence the quality and magnitude of anti-tumor adaptive immunity). In vivo imaging techniques are starting to provide some of these details [168]. In addition, peptide-, protein-, and cell-level knowledge can be encoded using computational tools, in the form of multiscale models, to aid in interpreting higher level observations, such as *in vivo *measurements.

## Translating Knowledge into the Clinic

In summary, cancer is a complex disease manifested by multiple changes in physiology distributed across a variety of time and length scales. In the previous sections, details associated with the role of IL-12 in tumor immunology have been described across these time and length scales. Variations within each of these levels propagate upward to reflect the variability in etiology of cancer and in clinical response to treatment at the patient level. Realization of individually tailored therapies requires identifying the underlying mechanistic basis for the clinical phenotype. A high degree of uncertainty is associated with determining such a mechanistic basis due to the limitations of experimental observation. Prior information obtained from preclinical studies, encoded in mathematical models, can be used to help interpret the limited information that can be obtained from the patients, as encouraged by the Food and Drug Administration [169].

In engineering parlance, this process is analogous to systems design, a complement to systems analysis. In systems design, our knowledge of the putative important components is used to assess how well mechanistic descriptions of these components recapitulate real system behavior. In immunology, a major hurdle for develop immunotherapies is integrating the knowledge obtained about individual molecules and cells to predict immune response [170]. In engineering, mathematics is used represent our knowledge of the components and simulation is used to create an expectation for how we expect the system to behave. An underlying theme in this review is the use of theory and simulation to build computational bridges across scales.

Recently, multiscale mathematical models have been used to help understand immunity to infectious pathogens [171], tumor invasion [172], receptor tyrosine kinase signaling [173], type 1 diabetes [174], and type 2 diabetes [175]. Integration of biological information across scales using multiscale models to predict clinical outcomes is an emerging field, described as systems medicine [176]. Despite these examples, one might suggest that building multiscale models is a futile exercise, given the uncertainty in the biological details associated with many of the time and length scales described here.

Yet, models play a central role in science [177]. One frequently creates a mental model of how one thinks a system behaves (i.e., a hypothesis) and creates a test (i.e., an experiment) to see whether the mental model is a valid representation of the system. The causal relationships implicitly encoded within a mental model are frequently depicted using a diagram or cartoon. Given the complexity of biological systems, mathematical models that incorporate mechanistic information provide value as they require an explicit statement of underlying assumptions and establish formal relationships between cause and effect. Creating a mechanistic model can also be useful in systems for which our knowledge is limited. Ultimately, mechanism-based mathematical models make predictions: what do we expect to happen in a particular system under particular conditions, given our current understanding of how the components of the system operate? If there is agreement between the observed data and the model predictions, the mechanistic model provides a causal explanation for the observed behavior. Conversely, differences between the expected behaviors and observed data identify areas where our understanding of the system is inadequate and reveal novel aspects of biology [118]. Thus, mathematical models extend our reasoning abilities by predicting the consequence of assumptions that may not be interpreted or understood through human intuition alone. This is analogous to experimental equipment, such as a flow cytometer, that extend human senses to observe phenomena [178].

## Conclusions

In closing, molecular targeted therapies have revolutionized the treatment of cancer. However, developing these drugs is challenging due to the frequent lack of clinical efficacy and emergent resistance. Shortcomings in the development of these compounds may be attributed to an inability to translate information among scales (e.g., how an in vitro assay correlates with clinical response). Understanding the relevance of scales is a central theme in science that transcends disciplinary boundaries [177]. This review was intended help educate readers to the diversity of time and length scales that underpin cancer pathophysiology. Interleukin-12 was used as an illustrative example to guide the reader through these concepts as it bridges innate to adaptive immunity and exerts potent antitumor activity. Thus, drawing attention to the diversity of time and length scales at work in a patient may improve our understanding of cancer and lead to the design of immunotherapies that are more effective.

## Competing interests

DJK holds stock from Entelos, Inc. The content is solely the responsibility of the author and has not been influenced by Entelos, Inc.

## Authors' information

DJK received his Ph.D. in Chemical Engineering from Northwestern University and is currently an Assistant Professor in the Department of Chemical Engineering and the Department of Microbiology, Immunology, and Cell Biology at West Virginia University. Prior to his current position, DJK developed multiscale disease models in the areas of atopic asthma, rheumatoid arthritis, type 1 diabetes, and type 2 diabetes for Entelos, Inc. (Foster City, CA http://www.entelos.com). Entelos is a life sciences company that, through predictive biosimulation, helps bring therapeutics to market faster.

## Authors' contributions

DJK conceived, drafted, finalized and approved the final manuscript.
